# Immune checkpoint inhibitors and Chimeric Antigen Receptor (CAR)-T cell therapy: Potential treatment options against Testicular Germ Cell Tumors

**DOI:** 10.3389/fimmu.2023.1118610

**Published:** 2023-02-13

**Authors:** Giuseppe Schepisi, Caterina Gianni, Maria Concetta Cursano, Valentina Gallà, Cecilia Menna, Chiara Casadei, Sara Bleve, Cristian Lolli, Giovanni Martinelli, Giovanni Rosti, Ugo De Giorgi

**Affiliations:** ^1^ Department of Medical Oncology, IRCCS Istituto Romagnolo per lo Studio dei Tumori (IRST) “Dino Amadori”, Meldola, Italy; ^2^ Unit of Biostatistics and Clinical Trials, IRCCS Istituto Romagnolo per lo Studio dei Tumori (IRST) “Dino Amadori”, Meldola, Italy

**Keywords:** germ cell tumors, GCT, chimeric antigen receptor, CAR-T, immune checkpoint inhibitors, testicular cancer, treatment

## Abstract

Germ cell tumors (GCTs) represent a heterogeneous neoplasm family affecting gonads and rarely occurring in extragonadal areas. Most of patients have a good prognosis, often even in the presence of metastatic disease; however, in almost 15% of cases, tumor relapse and platinum resistance are the main challenges. Thus, novel treatment strategies with both improved antineoplastic activity and minor treatment-related adverse events compared with platinum are really expected. In this context, the development and the high activity demonstrated by immune checkpoint inhibitors in solid tumors and, subsequently, the interesting results obtained from the use of chimeric antigen receptor (CAR-) T cell therapy in hematological tumors, have stimulated research in this direction also in GCTs. In this article, we will analyze the molecular mechanisms underlying the immune action in the development of GCTs, and we will report the data from the studies that tested the new immunotherapeutic approaches in these neoplasms.

## 1 Introduction

Germ cell tumors (GCTs) represent a heterogeneous neoplasm family affecting gonads and rarely occurring in extragonadal areas, such as retroperitoneum, mediastinum and pineal gland (1–3).

GCT is the most common neoplasm affecting young males between 15 to 44 years of age. The vast majority of patients have a good prognosis with high cure rates even in the presence of metastatic disease (4). However, in a small percentage of cases, GCTs deserve a poor prognosis with tumor relapse and resistance to platinum-based chemotherapy, and are treated with aggressive approaches including high-dose chemotherapy with support of hematopoietic progenitor cells (5–7). In addition, because of young age at diagnosis and high cure rate, many patients experience long-term survival related problems, including physical and psychosocial issues, most of which related to previous antitumor treatments, in particular chemotherapy (8–10). Thus, novel treatment strategies with both improved antineoplastic activity and minor treatment-related adverse events compared with platinum are really expected (11, 12). In this context, the development and the high activity demonstrated by immune checkpoint inhibitors in solid tumors and, subsequently, the interesting results obtained from the use of chimeric antigen receptor (CAR-)T cell therapy in hematological tumors, have stimulated research in this direction also in solid tumors, and more recently in GCTs (13). In this article, we will analyze the molecular mechanisms underlying the immune action in the development of GCTs, and we will report the data from the studies that tested the new immunotherapeutic approaches in these neoplasms.

## 2 Rationale for immunotherapy in GCTs

GCTs represent a highly curable neoplasm, but almost 15% of patients experienced recurrence; the exact mechanism of platinum resistance is not fully understood, but it is believed to have a multifactorial origin (14). One reason could be hided into relationship between GCT cells and surrounding tumor microenvironment (TME), which is currently under investigation.

Stromal cells and the extracellular matrix (ECM) can promote neoplastic proliferation and inhibit apoptosis mechanisms. In turn, cancer cells can influence TME activity (15, 16 Indeed, the latter is not constitutionally a protumoral environment, but surrounding conditions may transform an immune TME into a immune suppressive status and viceversa (17). In testis, microenvironment has an critical role during both developmental process and neoplastic transformation (18). The testis structure is divided into seminiferous tubules and interstitium. The testicular interstitial zone is composed of fibrocytes, androgen-producing Leydig cells, and immune cells, including lymphocytes, macrophages, mastocytes, natural killer, and dendritic cells (19–21).

Testis represents an immunologically privileged organ in mammals, because of its immune TME-mediate protection against autoimmune attack and its deficitary response to antigens. This “prerogative” is probably also involved in spermatogenesis and steroidogenesis mechanisms (22). In literature, spontaneous GCT regression cases are rarely reported, probably due to both the patient’s immune TME and the alteration in tumor vascularization (23). Moreover, GCT patients have been described to activate specific CD8+ and CD4+T cell-mediated immune responses against cancer/testis antigens. T cells are strongly present only in conjunction with the expression of these antigens, while they are much less numerous after treatment (24). However, it is currently unclear whether immunological privilege is implicated in GCT development (25). Several studies have been conducted in order to describe any difference in the immune response in case of GCT or GCT in situ: it has been reported that, comparing GCT with non-oncological testis diseases or in normal testis, T cells are ubiquitarious, whereas B cells and dendritic cells have been detected in GCT samples. Furthermore, pro-inflammatory cytokines, such as IL-6, IL-1β TNF-α), anti-inflammatory cytokines (TGF-β1), Th1-related cytokines (IFN-γ and IL-2), and chemokines (CXCL-13, CXCL-10, and CCL-5) were reported as strongly expressed only in GCTs (26). Notwithstanding, Hvarness et al. did not report any active immune surveillance in GCTs, with similar immune cell concentration in both GCT and normal testis samples (27). Recently, Skowron et al. analyzed the role of cross talk between GCT cells and TME, and demonstrated that this interaction stimulated the expression of ECM proteins, such as collagen I/IV and fibronectin, which in turn altered its structure, leading to a pro-tumoral TME. In those conditions, the researchers observed much more effective migration and adhesion properties in GCT cells as well as enhanced platinum resistance. The latter study suggests that targeting ECM (28) could become a novel therapeutic option, especially in relapsed GCT patients.

## 3 Prognostic biomarkers and potential new targets in GCTs

Among solid tumors, GCT represent an example of neoplasm without any significant mutational burden, as confirmed by The Cancer Genome Atlas (TCGA) (29). Several genome wide studies has been conducted in GCTs: in 4–31% of seminomas, and up to 14% of non-seminomas, driver mutations were detected in three genes (KRAS, NRAS and KIT) (30–33). Since their low incidence in GCT, a single universal mutation could not explain tumorigenesis in testis. So, it has been suggested a polygenic influence in GCT genesis and proliferation, involving an interaction among several susceptibility genes (up to 50 risk loci has been detected to date) (34). A recent study conducted in 137 GCT patients confirmed low mutation in the three known mutated genes (KIT, KRAS, and NRAS) and reported a frequency of 0.5 mutations per megabase (29). Similarly, to testicular GCTs, primary mediastinal GCTs also have low mutational burden, present mutations in RAS pathways, and since they are exclusively non-seminomatous tumors, KIT mutations are rare. However, unlike testicular GCTs, primary mediastinal tumors exhibit a higher percentage of p53 mutations (35).

GCT subtypes represent the developmental steps from embryonic stem cells toward more differentiated cells to somatic tissues. The mapping of GCT DNA-methylation status (methylome) clearly correlates with the state of differentiation in the GCT histotypes: Seminomas are typically unmethylated or severely hypomethylated tumors, Embryonal carcinomas demonstrate low to intermediate levels of global DNA methylation; well-differentiated yolk sac tumors and teratomas show high levels of DNA methylation (36). Therefore, this histological variability correlates both with the tumoral epigenetic heterogeneity, and with the epigenetic landscape of healthy tissues: in fact, hypermethylated pattern has been reported in differentiated somatic cells as well (37–39). Non-CpG methylation, acetylation, and methylation of histones are also involved in GCT development, but they are scarcely understood to date, instead of the microRNA (miR) signaling, which improved our knowledge about GCT molecular biology. The overexpression of pluripotency markers such as NANOG, OCT3/4 or a tissue stem cell factor KIT and its ligand are correlated with the unique GCT germline origin (40–42). Indeed, their expression has been linked to epigenetic regulation with both DNA methylation and histone acetylation (43–46).

In GCT, carcinoembryonic antigen claudin 6 (CLDN6), a tight junction associated membrane protein may represent an ideal chimeric antigen receptor (CAR) antigen because of its extracellular loop that can be targeted by T cells; moreover, it is silenced during organogenesis, thus is not expressed in healthy cells but only in various cancer cells, including GCT: indeed CLDN6 is expressed in approximately 93% of GCT (47).

## 4 Immune-related biomarkers in GCTs

Since the first new checkpoint inhibitors were approved in the oncology field, researchers have simultaneously begun to evaluate new immunological markers in different tumor histologies, including GCTs. Among these novel immune-related biomarkers, programmed-death receptor axis, including (i.e. PD-1 and its ligand PD-L1) was tested in GCT also; one immunohistochemistry study conducted by Fankhauser et al. confirmed its activation: indeed, a frequent PD-L1 expression in 479 GCT tissue samples was reported, regardless of the histological subtype (73% of seminoma and 64% of non-seminoma patients, respectively) (48). Analyzing data from TCGA database, a surrogate signature of “T-cell inflamed genes” was demonstrated in 47% GCT samples (49). Lobo et al. analyzed both CTLA4 and PD-L1 expression in GCTs: albeit they found high rates of CTLA-4 and PD-L1 expression in GCTs (96.3% and 85.5%, respectively), no significant correlations were demonstrated either between CTLA-4 expression and the GCT characteristics, such as IGCCCG grouping, rete testis or lymphovascular invasion, staging, nor between CTLA-4 intensity and recurrence-free survival (RFS) (p = 0.934). Instead, they demonstrated a PD-L1 expression in 24.9% of samples, with no significant differences between seminomas and non-seminomas, although PD-L1 resulted more frequent in choriocarcinomas than in teratomas. Curiously, they did not found any differences in terms of RFS among PD-1 positive and negative cases (50).

Another study applied a multiplicative quick score to evaluate PD-L1 expression in a semi-quantitative manner, demonstrating a correlation between scores and clinical outcome: in fact, significantly better PFS (HR=0.40; P =0.008) and OS (HR=0.43; P =0.040) were reported in GCT patients low PD-L1 expression levels (51). The predictive role of PD-L1 expression was also confirmed by Chovanec et al. The authors evaluated PD-L1 expression on tumor infiltrating lymphocytes (TIL) – whose prognostic role was previously demonstrated by Bols et al. (52) – and demonstrated that high PD-L1 expression on TIL was correlated with a significantly better prognosis than cases with lower levels (53). The same correlation was reported by Boldrini et al. (54) in the development of childhood malignant extracranial GCTs. Cheng et al. reported that PD-L1–positive TILs are detected in 85,9% of seminomas, 91% of embryonal carcinomas, 60% of yolk sac tumors 54,5% of choriocarcinomas, and 35.7% of teratomas (55). Shah et al. detected T-cell–inflamed TME, which is inversely correlated with AFP levels, more frequently in seminomas than in other GCT (49). Analyzing immune cells other than lymphocytes, similar differences in PD1 expression between seminomas and non-seminomas has been highlighted by other studies. Sadigh et al. detected PD-L1+ tumor-associated macrophages (TAM) rather in seminomas than non seminomas (56). Analyzing 22 types of immune TME, Song et al. demonstrated high expression of CD8+ T-cells, macrophages, and dendritic cells in GCTs compared with normal samples (57). Siska et al. reported an activated CD3+ T-cell infiltration, PD-L1 hyperexpression, and implemented PD-1/PD-L1 spatial interaction in seminomas; this characteristics were correlated with the better prognosis of this histotype, whereas high macrophage and neutrophil gene signatures were more frequently shown in nonseminomas. In both cases, decreased T-cell and NK-cell signatures,and elevated Treg, neutrophil, mast cell, and macrophage signatures were reported in advanced stage of GCTs (58).

More recently, in a Polish study conducted in a 180 GCT patient cohort,a correlation among 1) lower expression of immune checkpoint proteins V-domain Ig suppressor of T cell activation (VISTA) and PD-L1 on TME, 2) elevated inflammatory marker platelet-to-lymphocyte ratio and 3) higher risk of events was reported, suggesting an involvement of both local and systemic anti-tumor immune response in GCT**s** (59).

In spite of this, PD-L1 expression level does not seem to be predictive of response to immune-checkpoint inhibitors. This uncertainty in PD-L1 predicting response is quite common in several tumors. In fact, using PD-1/PD-L1 inhibitors, a significant response was more often reported in cases with PD-L1 expression, but some responses in PD-L1 negative tumors were described as well (60). To date, in GCT patients, PD-L1 expression in tumor and TIL correlates with an abundant immunogenic microenvironment but not with immunotherapy response. Perhaps, this incongruence could be only due to our incomplete knowledge on immune machinery.

A comprehensive molecular characterization conducted by Shen et al. did not demonstrated a significant neoantigen signal in GCT, so the disappointing results of immune check-point inhibitors in GCT could be partly due to very low mutational burden (29).

Two other studies evaluated the role of a systemic-immune infiltration index (SII), a marker of proinflammatory microenvironment, which is obtained from total neutrophils count, lymphocytes, and platelets. The first one evaluated several markers (i.e., low albumin and hemoglobin, high leukocytes, neutrophils, CRP, neutrophils to lymphocyte ratio, and SII) and demonstrated their correlation with poor prognosis in GCT (61). The second study confirmed a correlation between higher SII levels and poor prognosis in two independent GCT patient cohorts. The authors also evaluated the combined prognostic value of SII and PD-L1 expression on TIL, and reported a better prognosis in cases with low SII and high PD-L1 on TIL (62). Both the research group demonstrated the prognostic significance of SII regardless of the standard IGCCCG risk criteria (61, 62). Recently, these results were confirmed by Ribnikar et al. (63). Interestingly, poor prognosis in cases with high SII levels could be the prove that proinflammatory mechanisms stimulated by an aggressive tumor microenvironment represent the effect of an unsuccessful fight of the human immune system against the tumor progression (36). More recently, another study confirmed the prognostic role of SII in GCTs treated with high-dose chemotherapy (64).

Regarding other TME elements, Tumor Associated Fibroblasts (TAFs) have been shown to stimulate proliferation and metastasis in several tumor types (65–68). Indeed, TAFs secrete several soluble factors, which promote ECM to produce further soluble factors, including VEGF, HGF, TGFb, IL6, CXCL12, and CCL2 (69), which are involved in tumor proliferation. Moreover, GCT cells shown a significant miR-125b expression, which stimulates secretion of tumor-derived chemokines, such as CSF1 and CX3CL1, which in turn increase TAM recruitment (70). Other soluble factors, in particular IL-8, can lead to an increase in NF-kB and ABCB1, which are responsible of reduced Cisplatin sensitivity: this mechanism, described in gastric cancer cells, could be applicable to GCTs as well (71). It is therefore interesting to note that precisely the destruction of tumor cells mediated by platinum-based chemotherapy could at the same time stimulate the secretion of protumoral factors in the tumor stroma (72).

Another study evaluated the prognostic role of proinflammatory cytokines, like as IFN-α2, IL-2Rα, or IL-16, demonstrating their correlation with high risk clinical characteristics and poor survival in GCT (73). Another interesting cytokine is IL13RA2, strongly expressed in normal testicular cells and currently studied as a potential CAR target against other tumor types, such as glioblastoma multiforme (74).

Moreover, some researchers evaluated the prognostic role of β-1,4-galactosyl transferase-I (B4GALT1) in GCT. BAGALT1 is an enzyme involved in interaction and adhesion of immune cells; its role in disease control in stage I non-small cell lung cancer patients was reported by Lu et al. (75). Nilius et al. demonstrated that high B4GALT1 expression in peripheral T cells represents a marker of lower risk of relapse in GCT patients underwent salvage high-dose chemotherapy and peripheral stem cell transplant. The authors suggest that activated peripheral T cells may be crucial in cancer control. In fact, lectin stimulation of mononuclear cells with Concanavalin A determined a B4GALT1 upregulation from CD4+ T cells, which was correlated with IL-10 hyperexpression. The latter was in turn correlated with better outcome in GCT patients (76).

Hinsch et al. evaluated the immunohistochemic expression of T Cell immunoreceptor with Ig and ITIM domains (TIGIT) in 78 seminoma samples, and reported frequent expression of this immune checkpoint receptor, albeit with high variability in the relative prevalence of TIGIT+ and PD-1+ cells (77).

Two other promising therapeutic targets in immunotherapy are T-cell immunoglobulin and mucin domain-3 (TIM-3) and lymphocyte activation gene-3 (LAG-3): the first one is involved in T-cell exhaustion, which in turn could determine a failure of PD-1 monotherapy blockade or adaptive resistance to anti-PD-1 agents (78, 79). The second one is involved in immune homeostasis through an inhibition of T cell activation and cytokine secretion. In addition, higher LAG3 expression on TILs was correlated with higher PD-L1 expression (80). In spite of this, LAG3 and TIM3 expression in GCT cells were not higher than in nearby normal cells (81).

Recently, mismatch-repair (MMR) deficiency has been significantly related to PD-L1 expression, in different tumor types, including GCT (50). This deficiency makes the tumor more immune sensitive, and more prone to express higher levels of PD-L1. In GCTs, a correlation between MMR-deficiency, microsatellite instability (MSI) and platinum resistance was reported by Honecker et al. (82). More recently, the correlation between low MMR proteins expression and lower platinum sensitivity was confirmed in GCTs (83).

## 5 Immunotherapeutic approaches to GCT treatment

**Figure 1:**
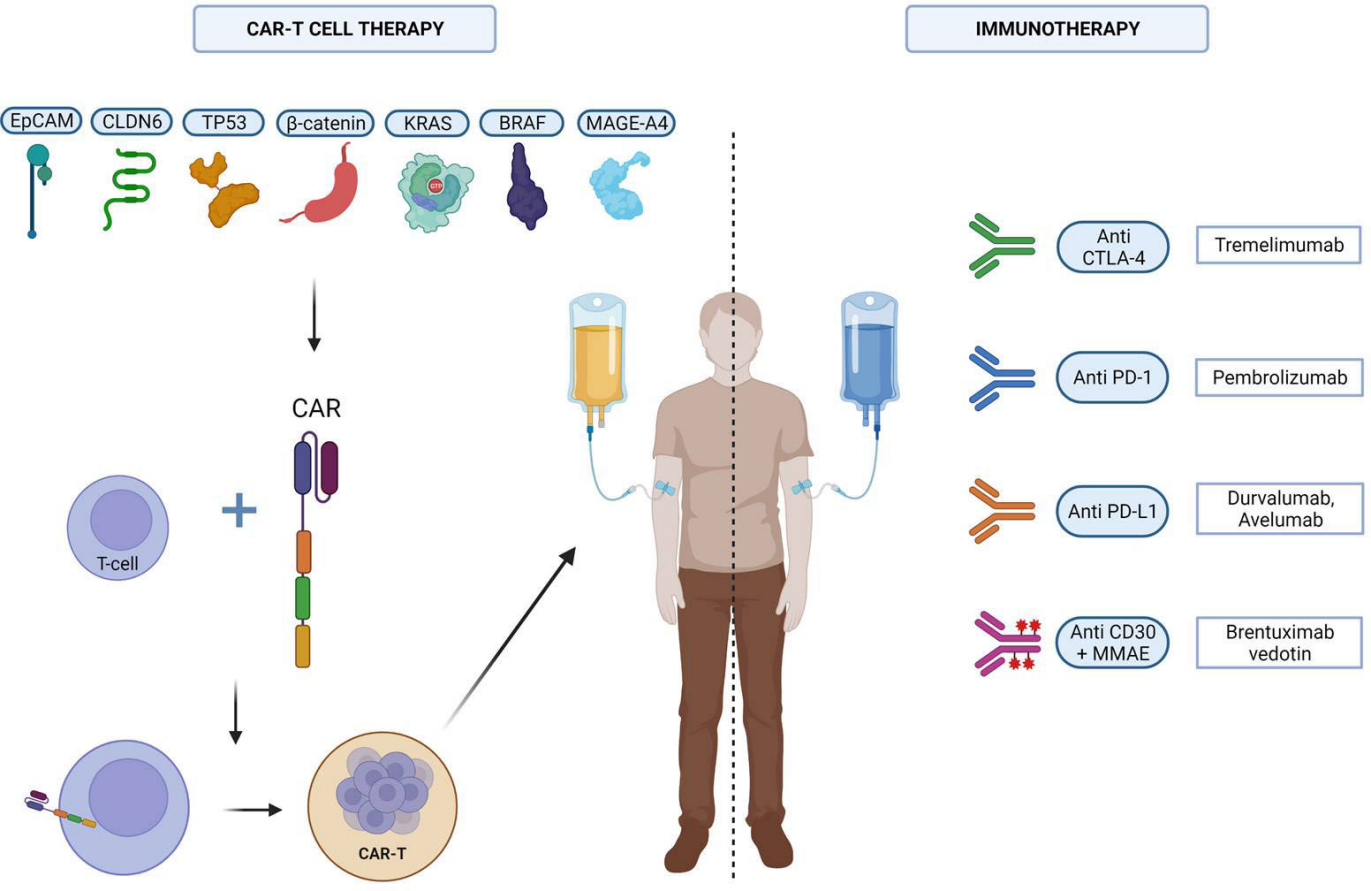
The different immunotherapeutic strategies tested against GCTs. Created with Biorender.

The potential immunotherapeutic strategy in GCT was investigated in several case reports and small patient cohorts (**Figure 1**).

### 5.1 PD1/PD-L1 axis

Most of the studies testing immunotherapeutic strategies against GCTs are based on PD-L1 checkpoint inhibitors (84, 85).

One trial reported a 33% of tumor volume regression based on RECIST version 1.1 and a 49% regression based on immune-related response criteria in a small cohort of embryonal cell carcinoma underwent a single dose of anti-PD-1 immune therapy (49). Zschäbitz et al. evaluated a series of seven platinum-refractory GCTs underwent high-dose chemotherapy and stem cell transplantation, and subsequently treated with anti-PD1 Nivolumab or Pembrolizumab. Four of them experienced rapid tumor progression and died after single-dose of immunotherapeutic drug. Only one PR was shown in one of the other three enrolled patients, but they underwent concomitant etoposide (86). Chi et al. reported a durable (radiographic and beta-HCG) response to Nivolumab in a pretreated poor risk metastatic choriocarcinoma (87). In another case report, one patient with choriocarinoma was treated with Pembrolizumab, but he experienced a rapid PD, thus immunotherapy was prematurely stopped (88). A phase II, single-arm trial (NCT02499952) tested anti-PD1 Pembrolizumab in 12 platinum-refractory GCT patients, enrolled regardless of PD-L1 expression. No OR was reported, with only SD in two patients for approximately 7 months and 5 months, respectively (89). Another phase II clinical trial (NCT02721732) was conducted in a small cohort of 12 GCT patients (10 men, 2 women) treated with Pembrolizumab. The drug was well tolerated. A SD was reported in 3 patients, but no OR was shown. The median PFS was 2.4 months and the median OS was 10.6 months (90).

Overall, these studies demonstrated a very limited antitumor activity of anti-PD-1 inhibitor monotherapy in GCTs (**Table 1**). Other researchers tested anti-PD-L1 inhibitors.

**Table 1:** Trials presenting anti-PD-1 inhibitor in GCTs.

Trial	Agent	Phase	Patients enrolled (n)	Status
NCT02499952 (88)	Pembrolizumab	II	12	Terminated (Lack of Efficacy)
NCT02721732 (90)	Pembrolizumab	II	12	Active, Not recruiting
NCT03403777 (91)	Avelumab	II	8	Terminated (lack of efficacy)
APACHE trial (92)	Durvalumab + Tremelimumab	II	22	Terminated (lack of efficacy)

A phase II clinical trial tested the PD-L1 inhibitor Avelumab in eight patients with relapsed/refractory GCT. Avelumab was well tolerated but with a limited activity in this small patient cohort (91). In another open label, phase II clinical trial, the PD-L1 inhibitor Durvalumab, alone or in combination with the anti-CTLA4 inhibitor Tremelimumab, was administered in a cohort of 22 GCT patients (11 underwent Durvalumab alone and 11 combination of both treatments). However, 72.7% of patient cohort treated with Durvalumab monotherapy experienced rapid PD, thus that arm was prematurely closed. One case of PR and one of SD was reported in the combination arm. PD-L1 expression was not correlated with tumor response (92).

### 5.2 Brentuximab vedotin

The poor results obtained with anti-PD1/PD-L1 inhibitors prompted researchers to focus on new immunological targets, for example on conjugated antibodies, such as brentuximab vedotin. This is an anti-CD30 antibody conjugate comprising a chimeric antibody bound to cell-surface antigen CD30 covalently conjugated to the cytotoxic antitubulin agent monomethylauristatin E. In a phase II study (NCT01461538), the researchers enrolled seven relapsed/refractory CD30-positive GCT patients, which underwent brentuximab-vedotin every 3 weeks. The authors reported two OR an one CR, which persisted for more than 4 years after four treatment cycles. In the other patient, a PR was demonstrated after 2 cycles, but after the fourth one, this patient experienced a rapid PD (93). In another study, Brentuximab Vedotin was administered in a cohort of 24 CD30-positive GCT patients. Eleven of them experienced a serum tumor markers reduction, whereas 11.1% of them reported a 3-month PFS and 85.7% of them 6-month OS. In a case report, a combination of Brentuximab Vedotin and Pembrolizumab in a highly pretreated patients with GCT led to a CR but at the cost of severe toxicities (grade 3 immune-mediated hepatitis, grade 3 polyneuropathy) (94).

### 5.3 CAR-T in GCTs

CAR-T cells are genetically engineered T cells, which display antigen-specific receptors on its external cell membrane. They are composed of four domains: (1) on the external extremity, a single-chain antibody fragment (scFV) also known as the antigen-binding domain; (2) a hinge region, which links scFV with the (3) transmembrane region; and (4) an intracellular region, which comprises the signal transduction part of the TCR, linked with one or two costimulatory domains (95).

Compared to other types of immunotherapy, CARs provide the following advantages: first of all, the immune mechanism of action depends on a surface–antigen interaction. Thus it is not MHC restricted, and that allows to use CAR-T cell strategy also in tumors without significant MHC expression (96). Moreover, The low antigen affinity in TCRs can determine off-target toxicities (97). In addition, CAR-T cells also provide T-cell lytic property (98).

To date, compared with haematological neoplasms, the road to using CAR-T cell therapy approach in solid tumors is much more complex; this is due to various problems, the most important being intra-tumor heterogeneity and TME-mediated protumoral activity (99). Indeed, no CAR-T cell therapy is approved in solid tumors.

Recently, four early-phase studies presented at ESMO Congress 2022 could represent a step forward in the development of CAR-T cell therapy into solid tumors (**Table 2**). Different approaches were tested: in two studies CAR-T cell therapies (100, 101) were used, a vaccine-targeted therapy in a third trial (102) and a T-cell receptor T-cell therapy in the fourth one (103), respectively. Among them, only one enrolled GCT patients (100).

**Table 2:** Ongoing clinical trials with adaptive cell therapies on solid tumors presented at ESMO 2022.

Trial	Agent	Target	Mechanism of Action	Phase	Patients enrolled/estimated enrolling
NCT04503278 (100)	BNT211	CLDN6	CAR-T	I	22 (13 testicular cancer)/96
NCT05028933 (101)	IMC001	EpCAM	CAR-T	I	7/48
NCT03953235 (102)	GRT-C903 GRT-R904	Version 1 (V1) neoantigens from KRAS, TP53, b-catenin, and BRAF. Version 2: only targeted KRAS neoantigens (G12C/D/V, Q61H).	Vaccine–targeted therapy +/- checkpoint inhibitors	I/II	26 (expected 144)
NCT04044859 (103)	ADP-A2M4CD8	MAGE-A4	T-cell receptor T-cell therapy +/- checkpoint inhibitors	I	29/90

BNT211-01 trial is a phase I first-in-human trial which tested BNT211, i.e. a CAR-T cell targeting CLDN6 both as monotherapy and in combination with a CLDN6-encoding CAR-T cell-amplifying RNA vaccine (CARVac) in patients with CLDN6-positive relapsed/refractory solid tumors (100). Twenty-two patients with GCT (n=13), ovarian (n=4), endometrial (n=1), fallopian tube (n=1), gastric cancer (n=1), sarcoma (n=1), and unknown primary (n=1) underwent CAR-T cell therapy at two different dose levels. 1 complete response (CR), 6 partial response (PR), 7 stable disease (SD) and 5 progressive disease (PD) were reported, with an overall response rate (ORR) of 33% (7/21) and a disease control rate (DCR) of 67% (14/21). Even more encouraging responses were observed in GCT cohort, with an ORR of 57% and a DCR of 85% (1 CR, 3 PR, 2 SD). A CR, in terms of both negative PET-CT scan and tumor markers was reported. Moreover, an abundant persistence of CAR-T cells was observed for >100 days, and in some cases for >200 days. Two patients had dose-limiting toxicities, including pancytopenia after lymphodepletion for the CAR T-cell monotherapy cohort, and hemophagocytic lymphohistiocytosis for the combination cohort, respectively. To date, the generation of CLDN6-based CAR-T cells has been switched to an automated process and dose escalation is ongoing (100).

## 6 Conclusion

In solid tumors, currently the most common immunotherapy is immune checkpoint blockade, which maintain the activity of T cells through the linkage with CTLA4 or PD1/PD-L1 molecules (104). The clinical response to these antibodies is generally more significant in tumors that carry a high mutational burden, albeit the response varies among single cases (105). CAR T-cell therapy is a novel fascinating strategy for non-responders to immune checkpoint inhibitors and for less immunogenic neoplasms, and demonstrated sensational results in some hematological tumors (106, 107). Curiously, in GCTs the current situation is the opposite: indeed, studies testing immune checkpoint inhibitors have led to disappointing findings, whereas CAR-T cell therapy showed promising results in a cohort of 13 GCT patients. This guarantees further investigation with a larger sample size. Should the role of CAR-T cell therapy in GCTs be confirmed, this would represent a significant step forward in this category of patients who are so young, for whom only chemotherapy is currently approved. Furthermore, young age should represent an element in favor of good tolerance to treatment, regardless of any known side effects related to CAR-T cell therapy (95). Furthermore, a better understanding of the mechanisms of activation of the immune system, including epigenetic influence in immune checkpoint expression (108), could also lead to a reconsideration of therapy with immune checkpoint inhibitors against GCTs.

## Author contributions

Conceptualization: GS and UD. Methodology: GS, CG, UD. Validation: UD. Writing original draft preparation: GS. Writing—review and editing: UD, CG and GS. Supervision: UD, CG, MC, CC, SB, GM, GR. All authors contributed to the article and approved the submitted version.
